# Long-term progestin contraceptives (LTPOC) induce aberrant angiogenesis, oxidative stress and apoptosis in the guinea pig uterus: A model for abnormal uterine bleeding in humans

**DOI:** 10.1186/2040-2384-2-8

**Published:** 2010-04-27

**Authors:** Graciela Krikun, Irina A Buhimschi, Martha Hickey, Frederick Schatz, Lynn Buchwalder, Charles J Lockwood

**Affiliations:** 1 Department of Obstetrics, Gynecology and Reproductive Sciences, Yale University School of Medicine, New Haven, CT, 06510, USA; 2 School of Women's and Infants' Health, University of Western Australia, Subiaco, WA 6008, Australia

## Abstract

**Background:**

Irregular uterine bleeding is the major side effect of, and cause for, discontinuation of long-term progestin-only contraceptives (LTPOCs). The endometria of LTPOC-treated women display abnormally enlarged, fragile blood vessels (BV), decreased endometrial blood flow and oxidative stress. However, obtaining sufficient, good quality tissues have precluded elucidation of the mechanisms underlying these morphological and functional vascular changes.

**Methods:**

The current study assessed the suitability of the guinea pig (GP) as a model for evaluating the uterine effects of LTPOC administration. Thus GPs were treated with a transdermal pellet for 21 days and examined for endometrial histology, angiogenic markers as well as markers of oxidative stress and apoptosis.

**Results and Discussion:**

We now demonstrate that GP uteri were enlarged by both estradiol (E2) and medroxyprogesterone acetate (MPA) (p < 0.001). Effects of MPA on uterine weight differed significantly depending on E2 levels (p < 0.001), where MPA opposed the E2 effect in combined treatments. Angiogenesis parameters were similarly impacted upon: MPA alone increased BV density (p = 0.036) and BV average area (p = 0.002). The presence of E2 significantly decreased these parameters. These changes were associated with highly elevated of the lipid peroxidation product, 8-isoprostane (8-isoP) content in E2+MPA-treated and by nuclear 8-OH-deoxyguanosine (8oxoG) staining compared to all other groups (p < 0.001). Abnormalities in the E2+MPA group were consistent with chromatin redistribution, nuclear pyknosis, karyolysis and increased apoptosis as observed by a marked increase in TUNEL labeling.

**Conclusions:**

LTPOC exposure alters endometrial vascular and tissue morphology consistent with oxidative stress and apoptosis in a complex interplay with endogenous estrogens. These findings are remarkably similar to *in vivo *change observed in the human uterus following LTPOC administration. Hence, the GP is an excellent model for the study of LTPOC effects on the uterus and will be extremely useful in determining the mechanistic pathways involved in this process which cannot be conducted on humans.

## Introduction

Because of their safety and efficacy, long-term progestin-only contraceptives (LTPOCs) are well-suited for women with restricted access to health care or in whom estrogen containing contraceptives are contraindicated. Unfortunately, administration of LTPOCs leads to irregular uterine bleeding in the majority of users [1,2]. Such bleeding disturbances are the primary indication for discontinuation of therapy[1,2].

Endometria from LTPOC-treated patients display dilated, thin walled, fragile vessels that are irregularly distributed across the endometrial surface [3-5]. Previous studies from our laboratory [6,7] as well as others [8-14] demonstrated that LTPOC therapy produced a statistically significant increase in mean lumen diameter of microvessels at bleeding versus non-bleeding sites [7] and that the key regulators of endometrial angiogenesis, vascular endothelial growth factor (VEGF) and angiopoietin-2 (Ang-2) were up-regulated in endometria treated with LTPOC[6,9]. Moreover, we demonstrated that hypoxia and reactive oxygen species (ROS) induced aberrant angiogenesis by reducing endometrial blood flow, inducing hypoxia, decreasing the ratio of the angiostatic agent, angiopoietin-1 to the angiogenic factor, angiopoietin-2 [3,5,6].

While past studies produced descriptive information regarding the possible causes of abnormal uterine bleeding following LTPOC treatment, considerations of difficulty in attaining good quality tissues from humans preclude functional studies of the mechanistic pathways involved in this process. Poor understanding of the mechanisms underlying bleeding has limited the development of effective therapies for abnormal bleeding with LTPOC. To further understand these mechanisms, we determined whether the guinea pig (GP) was a relevant model to study the uterine effects of LTPOC administration. The GP was chosen because its endometria display functional estrogen and progesterone receptors [15] as well as other properties closely related to the humans including spontaneous estrous cycling and hemochorial placentation [16-19]. In order to elucidate mechanisms underlying LTPOC-induced abnormal uterine bleeding, we evaluated the separate and interactive effects of estrogen and progestin on GP-endometrial weight, vascular morphology, oxidative stress and apoptosis.

## Materials and methods

### Guinea pigs

Eighteen nulliparous female GPs, aged 2-6 months, were subjected to bilateral oopherectomy and then given subcutaneous implants of 50 mg medroxyprogesterone acetate (MPA)-cholesterol-based 21 day time-release pellets or 5 mg estradiol (E2) cholesterol based 21 day time-release pellets (Innovative Research of America; Sarasota, FL) or both. Thus, animals received the treatment as follows: MPA (n = 6), E2 (n = 6), E2+MPA (n = 3) or placebo (n = 3). After three weeks, hysterectomy was performed and the right uterine horn was formalin fixed whereas the left horn was snap frozen for subsequent studies. These studies were approved by both Charles River (Wilmington, MA) and Yale University IACUC offices.

### Histology

The specimens were weighed and then formalin fixed, and paraffin embedded. Five micron sections were cut and stained with Hematoxylin-Eosin or Trichrome Mason (Sigma-Aldrich, St. Louis, MO) by conventional histological procedures as described [20] or used for immunohistochemistry as illustrated below.

### Immunohistochemistry

Sections were stained for von Willebrand factor (vWF) with the AB6994 primary antibody (Abcam, Cambridge, MA) at 1-10,000 dilution or 8-oxoG with the x 24326 primary antibody at 1-100 (Oxis International, Foster City, CA). For negative controls, normal IgG isotypes which were derived from the animals from which the antibodies were prepared and used at the same concentrations as the primary antibody. The sections were washed and the appropriate secondary biotinylated antibody (Vector Laboratories, Inc., Burlingame, CA, USA) was added per the manufacturer's instructions.

The antigen-antibody complex was detected with 3,3'-diaminobenzidine with or without nickel sulfate as the chromogen solution (Vector Laboratories). When nickel sulfate was used, no hematoxylin counterstaining was conducted. For each condition, 3 different slides were assessed and at least three independent areas of each slide photographed. For vWF, 6 randomly selected fields were digitally captured at 200× magnification using an Olympus microscope with digital camera. Vessel size, density and heterogeneity were measured in each field by computerized selection of the stained vessels with the public domain image analysis software Image J as previously described by others [21,22]. All steps including field acquisition and vessel morphology measurement were performed blinded. For the other endpoints immunostaining intensities were ascertained with Image-J.

### Lipid peroxidation profile

The isoprostanes are a family of eicosanoids of non-enzymatic origin produced by the random oxidation of tissue phospholipids by oxygen radicals [5]. The levels of 8-IsoP were measured in all 4 treatment groups on the frozen uterine horn obtained as described above. The samples were sonicated in 0.1 M Tris (pH 7.4) and diluted 1:5 in eicosanoid affinity buffer (Cayman Chemical Company, Ann Arbor, MI) and 8-isoP was detected by ELISA as we previously described [5]. The sensitivity of this ELISA is 10 pg/ml. Limit of detection: 80% B/B0: 2.7 pg/ml

### Apoptosis

Assessment of apoptosis was conducted on formalin-fixed, paraffin-embedded tissues with ApoTag peroxidase labeling kit (Chemicon International, Temecula, CA) as per the manufacturer's instructions. The procedure is based on detection of free 3'OH DNA termini *in situ*. To assess differences in apoptotic levels, photographs were taken from 3 representative areas of each slide at x200 magnitude under identical camera settings. The slides were analyzed with Image J by setting the minimum threshold that allowed for the visualization of the stained nuclei only. This threshold was maintained throughout the analysis of all subsequent slides. All values were subjected to analysis with Sigma Stat (Systat Software Inc, Chicago, ILL) utilizing the recommended ANOVA provided by the program based on sample distribution. The mean particle stained average +/- standard error of the mean were then represented by bar graphs.

### Statistical Analysis

Statistical analysis was conducted by ANOVA using the Sigma Stat Program (SPSS Inc., Chicago, IL).

## Results

### Morphology

**Figure 1:**
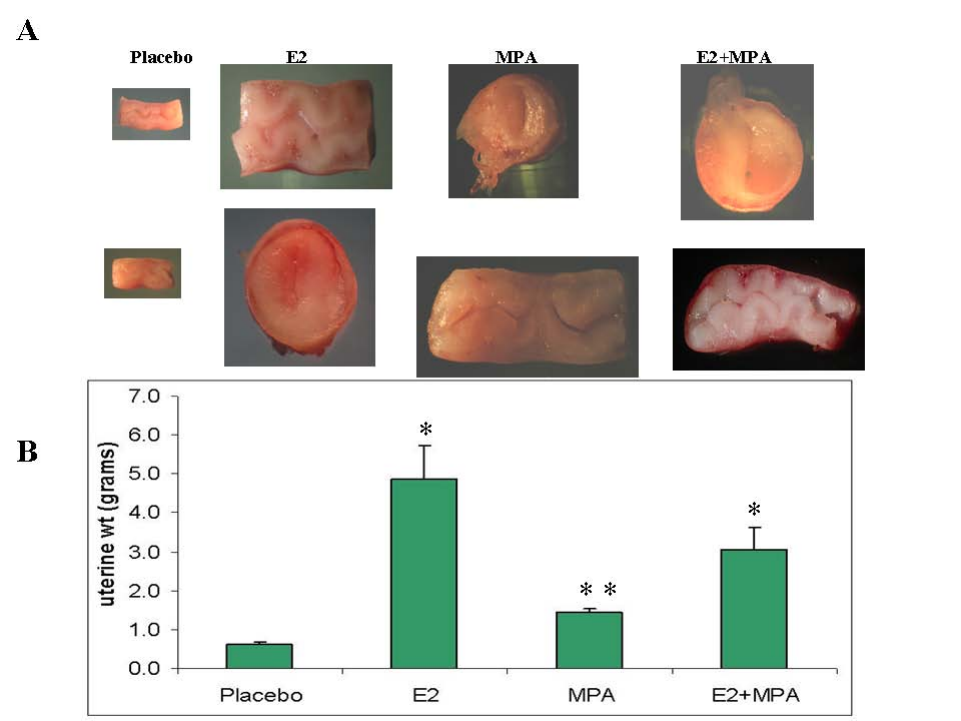
**Gross morphological analysis**. A) Transverse and longitudinal sections of GP uteri following treatment with placebo, E2, MPA or E2+MPA as described in Methods. B) Bar graph represents the mean +/- standard error of the mean for 6 experiments as determined by ANOVA followed by Student-Newman-Keuls test. * p < 0.01, ** p = 0.25

Figure 1 demonstrates that E2 (p < 0.001), MPA (p < 0.02) and E2+MPA (p < 0.05) all increase uterine wet weight compared with controls. The greatest effects were observed with E2 treatment, consistent with the proliferative effects seen in human endometrial following estrogen-only treatment. As expected, the effects of MPA on uterine weight differed significantly depending on E2 levels, where MPA opposed the E2 effect in combined treatments.

**Figure 2:**
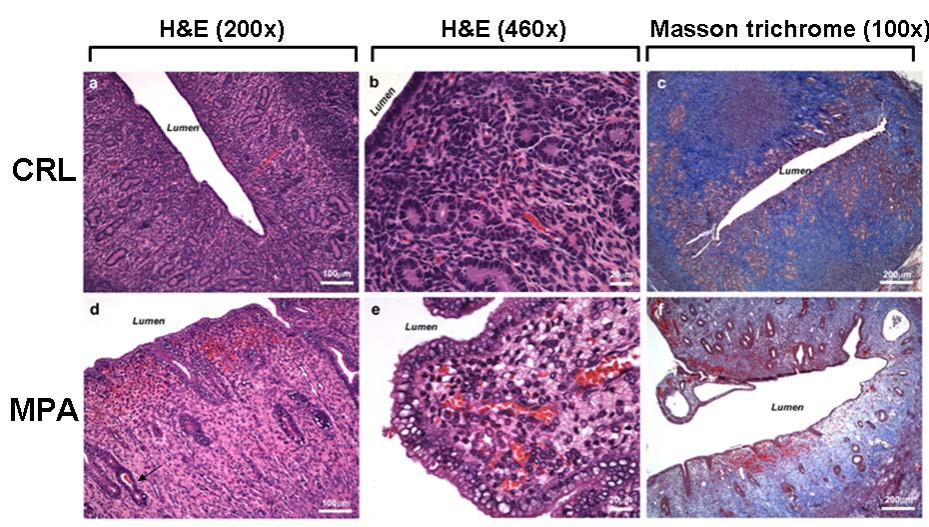
**Histological analysis**. Samples were stained with H&E or Mason Trichrome as described in Methods. Controls (CRL: Panels a-c). Sub-endometrial edema is observed in MPA-treated animals (Panels d-f).

Histological analysis of the samples was conducted after fixation and tissues were cut at 5 μ. Samples were stained with H&E or Mason Trichrome (Figure 2). Sub-endometrial edema is observed in MPA-treated animals.

### Angiogenic parameters

**Figure 3:**
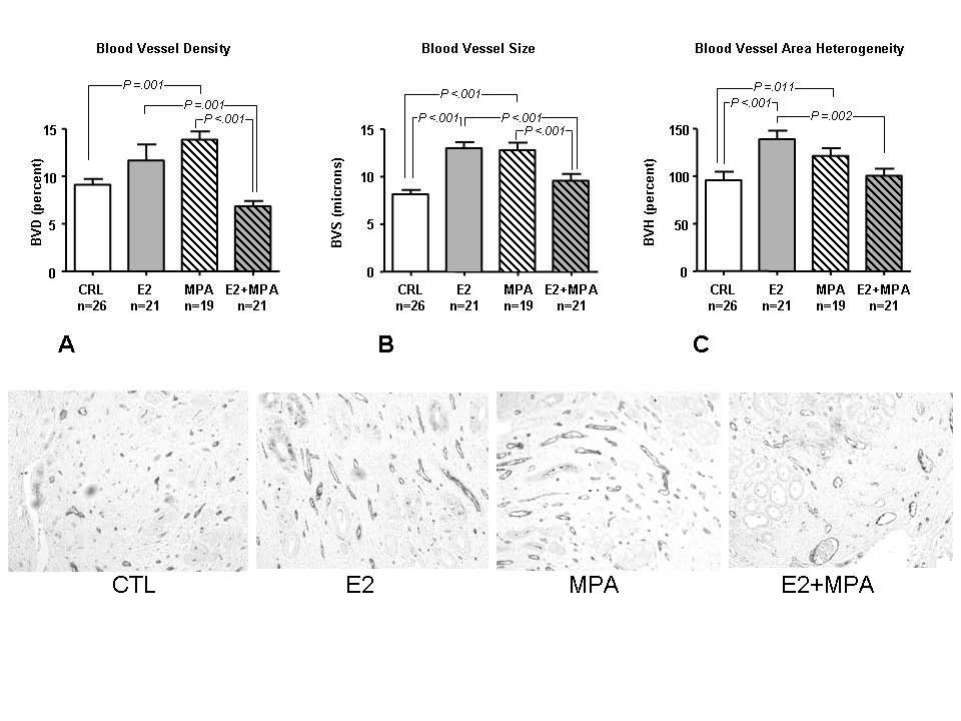
**Vascular morphology: (*Top*) vWF staining was conducted on formalin fixed, paraffin embedded tissues for placebo.** Bar graphs represents the mean +/- standard error of the mean for placebo control (CTL), E2, MPA or E2+MPA. **A) **Average blood vessel density, **B) **blood vessel size and **C) **blood vessel area heterogeneity. Statistical analysis were conducted by a two way ANOVA followed by Student-Newman-Keuls test for the 4 treatment groups. (*Bottom*) From left to right, representative IHC of vWF for CTL, E2, MPA and E2+MPA (20×).

Figure 3 displays staining for the endothelial marker, vWF in endometria treated with placebo, E2, MPA or E2+MPA. Based on that staining, the following endpoints were analyzed as follows: Blood vessel density (BVD) = area occupied by blood vessel lumen/area analyzed × 100. Blood vessel size (BVS) = average blood vessel diameter for the field analyzed (microns) and Blood vessel heterogeneity (BVH) = relative SD of blood vessel area for the field analyzed (SD/average area × 100).

Thus, angiogenic parameters were impacted upon as follows: MPA alone increased BV density (p = 0.036) and BV average area (p = 0.002). The presence of E2 significantly decreased these parameters (BV density mean SEM: CRL: 9.4 1.0%, E2: 10.3 1.6%, MPA: 13.6 1.1%, E2+MPA: 6.0 0.7%, p = 0.002).

### Oxidative stress

**Figure 4:**
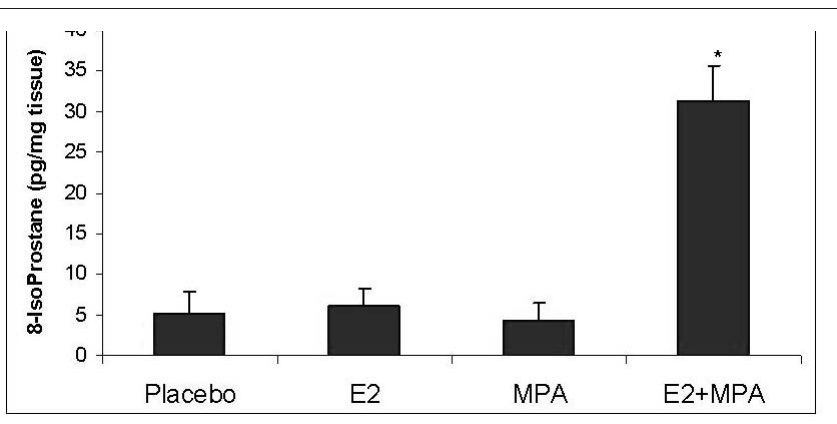
**Lipid peroxidation profile**. The levels of 8-IsoP were measured in all 4 treatment groups as described in Methods. Bar graph represents the mean ELISA values +/- standard error of the mean. Statistical analysis were conducted by one way ANOVA with Student-Newman-Keuls post hoc test (n = 6, *p < 0.001)

**Figure 5:**
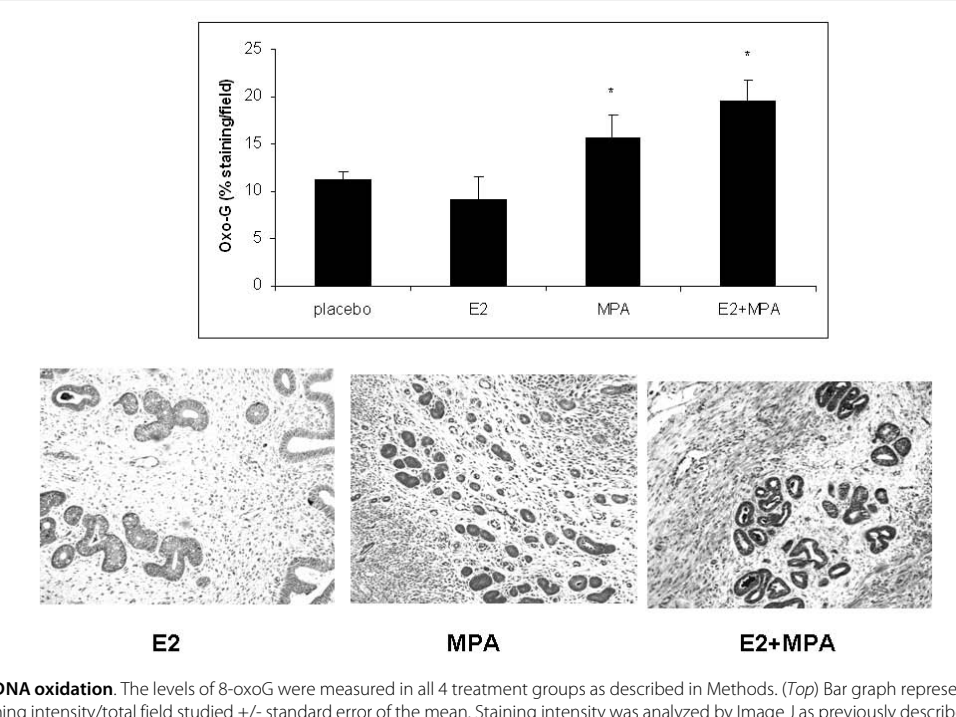
**DNA oxidation**. The levels of 8-oxoG were measured in all 4 treatment groups as described in Methods. (*Top*) Bar graph represents the average staining intensity/total field studied +/- standard error of the mean. Staining intensity was analyzed by Image J as previously described [21,22]. Statistical analysis were conducted by one way ANOVA with Student-Newman-Keuls post hoc test (n = 6, *p < 0.001 compared to E2). (*Bottom*) From left to right, representative IHC of 8oxoG for E2, MPA and E2+MPA (20×).

Levels of 8-isoprostane (8-IsoP) production were evaluated in endometrial extracts obtained from uteri treated with the various steroids. Figure 4 demonstrates that an eight-fold elevation in 8-IsoP levels occurred in uteri derived from E2+MPA-treated animals compared to all other groups (p < 0.001). Figure 5 displays immunohistochemical staining for 7,8-dihydro-8-oxoguanine (8-oxoG) which reflects ROS damage to DNA [23]. It is important to note however, that this endpoint alone is not conclusive of oxidative damage but also necrosis. That is why the study includes one of the best endpoints for oxidative stress which is 8-isoP (see above)

Expression of 8-oxoG was significantly higher in E2+MPA treated groups compared to E2 alone. A significant, though diminished effect was observed with MPA alone. However, no statistical differences were observed between E2 and the placebo control.

### Apoptosis

**Figure 6:**
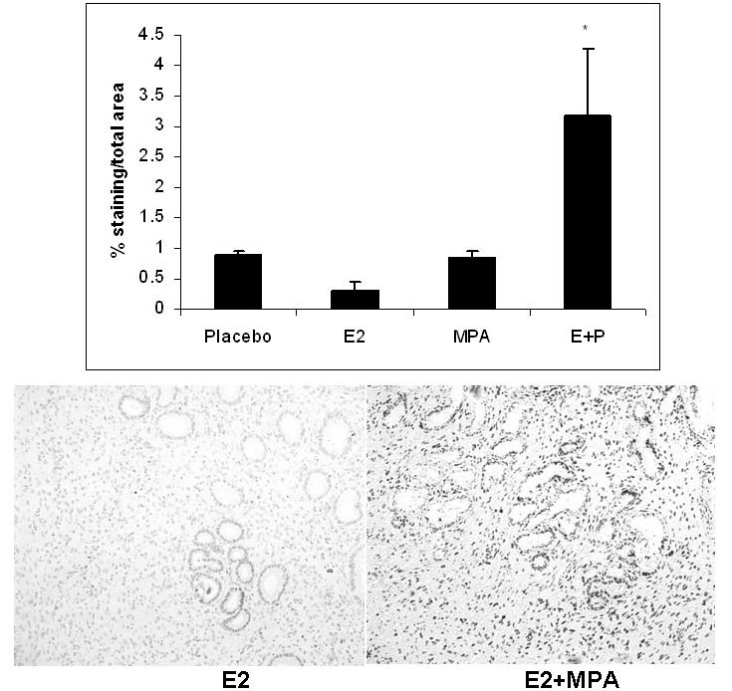
**Apoptosis: The apoptotic index was established for all 4 treatment groups after staining nuclei with Apotag as described in Methods**. The bar graph represents the average staining intensity/total field studied +/- standard error of the mean following analysis with Image J. Statistical analysis were conducted by one way ANOVA with Student-Newman-Keuls post hoc test (n = 3,*p < 0.05).

In humans, LTPOC treatment results in enhanced apoptosis of the endometrial glands and stroma [24]. Figure 6 demonstrates a similar apoptotic profile of E2+MPA group as reflected by increased TUNEL labeling. By contrast, MPA did not displayed statistical differences in TUNEL labeling compared to E2 or placebo.

## Discussion

Endometria from LTPOC-treated patients display dilated vessels that are irregularly distributed across the endometrial surface [3-5]. These dilated vessels bleed on minimal pressure and have deficient vascular basement membrane components [25,26] In the present studies we examined the effects of hormone treatment on a guinea pig LTPOC model. Because they undergo estrous cycling, the guinea pig more closely emulates the reproductive system of humans [27,28] compared to other rodents.

These studies demonstrate that treatment of guinea pigs with progestin and in particular with E2+MPA resulted in changes in endometrial vascular morphology, as well as increased markers of apoptosis and oxidative stress similar to that observed in human LTPOC-treated endometria. In humans, LTPOC treatment occurs in the setting of continuous low-level ovarian-derived estrogen production. Since the GPs used in the current study are ovariectomized, the E2+MPA treated animals are likely to most closely resemble LTPOC-treated humans.

In prior studies, we have demonstrated that LTPOC results in both reduced endometrial blood flow and increased oxidative stress [5,6]. These findings are associated with immunohistochemical evidence of increased angiogenic factor production. Further, hypoxia and oxidative stress induce increased production of vascular endothelial growth factor (VEGF) and reduced production of the angiostatic agent, angiopoietin-1 in cultured human endometrial stromal cells [4,6,9]. Hypoxia and oxidative stress also greatly enhance production of the highly angiogenic molecule, angiopoietin-2, in cultured endometrial endothelial cells [29]. However, it is unclear how LTPOCs exert the initial vasoconstrictive effects on human endometrium. The availability of this animal model should allow dissection of the underlying mechanism driving this vascular phenomenon. Information gleaned from the experimental results presented in this study are expected to ultimately improve the formulations and acceptability of LTPOC therapies by reducing aberrant angiogenesis and related irregular, unpredictable bleeding.

## Competing interests

The authors declare that they have no competing interests.

## Authors' contributions

GK and CL conceived and designed the experiments and wrote the manuscript. IB, FS and LB carried out the ELISAs and immunohistochemical procedures and statistical analysis. MH contributed in the critical analysis of the paper.
